# Artificial Intelligence in Psychiatry: A Review of Biological and Behavioral Data Analyses

**DOI:** 10.3390/diagnostics15040434

**Published:** 2025-02-11

**Authors:** İsmail Baydili, Burak Tasci, Gülay Tasci

**Affiliations:** 1 Vocational School of Technical Sciences, Fırat University, 23119 Elazığ, Türkiye; ibaydili@firat.edu.tr; 2 Department of Psychiatry, Elazığ Fethi Sekin City Hospital, 23280 Elazığ, Türkiye

**Keywords:** artificial intelligence, psychiatry, EEG, ECG, natural language processing, social media, explainable AI, biomarkers, mental health, machine learning, large language models

## Abstract

Artificial intelligence (AI) has emerged as a transformative force in psychiatry, improving diagnostic precision, treatment personalization, and early intervention through advanced data analysis techniques. This review explores recent advancements in AI applications within psychiatry, focusing on EEG and ECG data analysis, speech analysis, natural language processing (NLP), blood biomarker integration, and social media data utilization. EEG-based models have significantly enhanced the detection of disorders such as depression and schizophrenia through spectral and connectivity analyses. ECG-based approaches have provided insights into emotional regulation and stress-related conditions using heart rate variability. Speech analysis frameworks, leveraging large language models (LLMs), have improved the detection of cognitive impairments and psychiatric symptoms through nuanced linguistic feature extraction. Meanwhile, blood biomarker analyses have deepened our understanding of the molecular underpinnings of mental health disorders, and social media analytics have demonstrated the potential for real-time mental health surveillance. Despite these advancements, challenges such as data heterogeneity, interpretability, and ethical considerations remain barriers to widespread clinical adoption. Future research must prioritize the development of explainable AI models, regulatory compliance, and the integration of diverse datasets to maximize the impact of AI in psychiatric care.

## 1. Introduction: The Rise of Artificial Intelligence in Psychiatry

Medical diagnostic processes are vital in mental health, as in all fields of medicine. However, this process stands out as particularly problematic due to its complexity and subjective elements. Psychiatric diagnosis involves associating observed symptoms in patients with specific disease categories [1]. These symptoms represent the collected information during the assessment, while the diseases describe the abnormalities in these symptoms [2]. Over time, advancements in scientific medicine have more systematically defined diseases through causal chains and systems such as the International Classification of Diseases, structuring the diagnostic process more systematically [3]. Yet, these systems have not eliminated the error margin, increasing the need for technological solutions.

Artificial intelligence (AI) enables computers to mimic specific functions of human intelligence such as learning, reasoning, decision-making, and problem-solving [4,5,6]. Today, AI’s potential to revolutionize medicine and health services is noteworthy [7]. In particular, AI’s ability to analyze large volumes of data to provide accurate and fast results enhances the efficiency of clinical processes while reducing the workload of health professionals. In today’s world, where health expenses are rising and chronic diseases necessitate more frequent follow-ups, the focus on individuals’ quality of life is intensifying. Consequently, the solutions provided by AI are becoming increasingly important [8].

Psychiatry is a discipline known for its difficulty and complexity within the health sciences [9]. The diagnosis of psychiatric disorders typically relies on subjective assessments and clinical observations [10]. However, these processes often take a long time to reach an accurate diagnosis and establish an effective treatment method, requiring high expertise. These limitations in traditional approaches increase the importance of integrating AI-supported methods into psychiatry [11]. In particular, AI’s capabilities in data analysis, modeling, and prediction offer a revolutionary transformation by providing more objective and reliable results in psychiatric evaluation processes.

The rise of AI in the health field has been made possible by technological advancements and the applicability of machine learning (ML) methods to health data [12,13,14]. Initial steps were taken in the second half of the 20th century with the use of statistical methods and algorithms. The introduction of ML techniques into the health sector in the 1990s laid the groundwork for AI’s integration into psychiatry, and during this period, clinical decision support systems began to be developed. From the 2000s onwards, methods allowing for big data analysis and computers with high processing capacities have become widespread, making AI applications more sophisticated. Today, advanced AI technologies such as deep learning, natural language processing, and neural networks are actively used in diagnosing psychiatric disorders, analyzing symptoms, and personalizing treatment processes.

**Figure 1:**
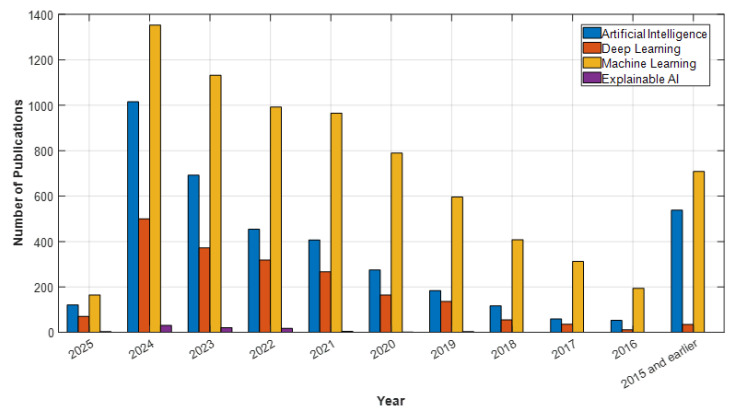
ScienceDirect Data: Psychiatry and AI Topics.

**Figure 2:**
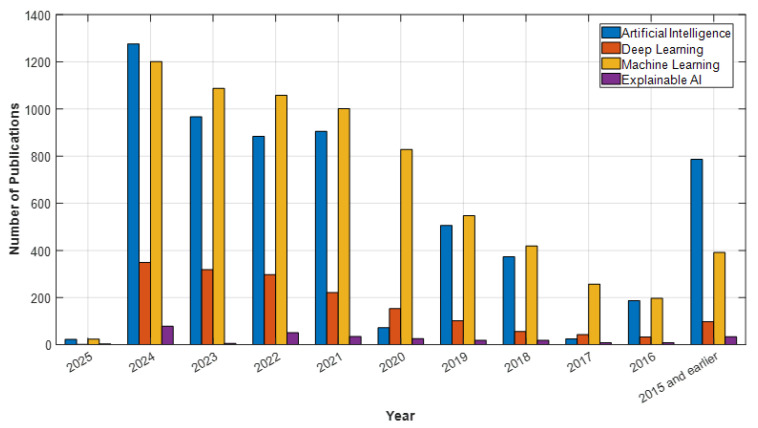
PubMed Data: Psychiatry and AI Topics.

**Figure 3:**
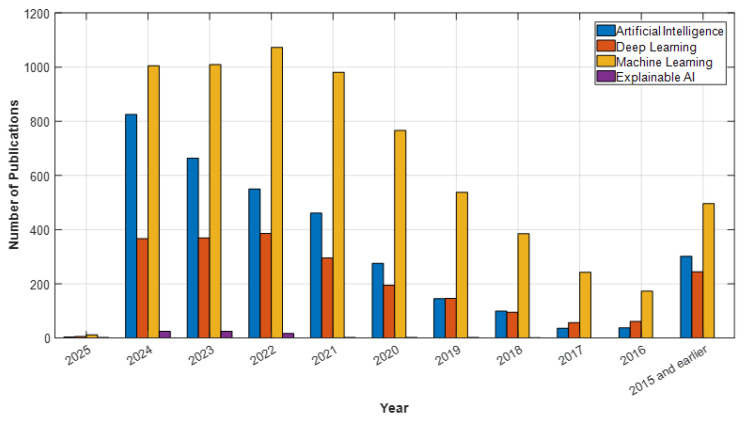
WOS Data: Psychiatry and AI Topics.

**Figure 4:**
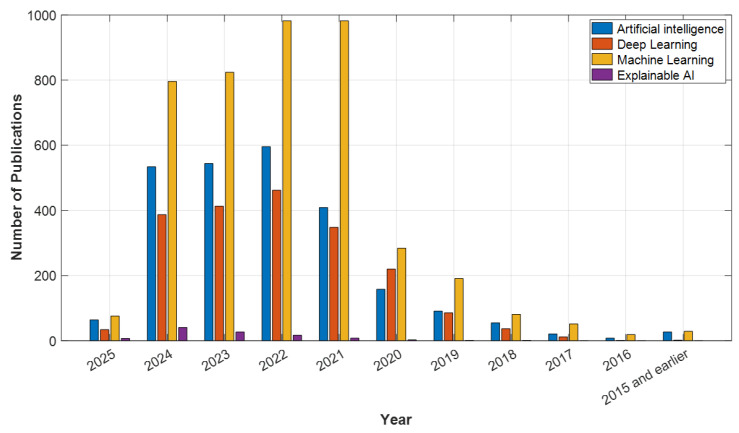
MDPI Data: Psychiatry and AI Topics.

In light of these developments, AI is positioned not only as a clinical decision support tool but also as a solution that will enhance access to mental health services. Understanding the historical development and current applications of AI in psychiatry is crucial. This article will thoroughly examine the historical development of AI in psychiatry, its current applications, and its potential future impacts. The goal is to broadly evaluate the opportunities AI presents in psychiatry, the challenges encountered, and the ethical dimensions, thereby highlighting the contributions this technology can make to the field’s future. According to data from Web of Science (WOS), PubMed, ScienceDirect, and open-access publishers like MDPI, studies on artificial intelligence, deep learning, machine learning, and explainable AI in psychiatry have shown a significant increase in recent years. In particular, after 2020, the number of articles published in these fields has surged, reaching a peak in 2024. For example, in 2024, WOS published 1005 articles, PubMed 1276 articles, ScienceDirect 1353 articles, and MDPI 796 articles on machine learning, making it the most studied topic of the year (see Figure 1, Figure 2, Figure 3 and Figure 4). Under the category of artificial intelligence, a total of 6004 articles were published in PubMed, 3400 in WOS, 3915 in ScienceDirect, and 2517 in MDPI. Although explainable AI has fewer studies compared to other areas, there has been a notable increase by 2024, particularly with 78 articles in PubMed and 25 in WOS. Although the year 2025 has only recently begun, the number of early-access articles across all databases indicates that these fields are still on the rise. The data demonstrate that AI technologies are increasingly gaining academic and clinical interest in psychiatry and hold significant research potential in these areas.

## 2. Methodology

This review adopts the narrative review framework to explore the burgeoning intersection of AI and psychiatry. This approach was selected due to its suitability for synthesizing broad conceptual and methodological landscapes prevalent in nascent but rapidly evolving fields. The narrative method facilitates a comprehensive examination of diverse research outputs encompassing EEG, ECG, natural language processing (NLP), and AI’s application in analyzing social media data for psychiatric insights. The literature search was executed through an extensive examination of databases such as Web of Science (SCIE index), PubMed, and ScienceDirect. The search strategy was carefully crafted using a combination of keywords: ‘artificial intelligence AND psychiatry’, ‘EEG AND AI’, ‘ECG AND mental health’, ‘NLP AND psychiatry’, and ‘social media AND mental health surveillance’. This strategy aimed to encapsulate the multidisciplinary nature of AI applications across various facets of psychiatric research. The inclusion criteria mandated that studies must provide empirical data or substantial theoretical analysis on the application of AI within psychiatric settings. Articles were considered if they were published in peer-reviewed journals and were written in English. The exclusion criteria were set to omit articles not peer-reviewed, such as conference abstracts and opinion pieces, as well as studies that did not focus explicitly on AI applications in psychiatric contexts. Data from selected articles were meticulously extracted and included the following information: authors, year of publication, study aims, AI technology utilized, primary outcomes, and their implications for psychiatric practice. These data served as the foundation for a narrative synthesis aimed at threading together thematic consistencies and divergences across the selected studies. The synthesis process involved categorizing the articles according to the type of AI technology employed and its application within psychiatric practice. This categorization facilitated a detailed thematic analysis, allowing for an in-depth discussion of technological advancements, application challenges, and the potential trajectory of AI in psychiatry. The rigor of the selected studies was critically assessed based on their methodological soundness, the robustness of findings, and the prestige of the publication outlets. Such a critical appraisal was pivotal in ensuring that the conclusions drawn from the review were grounded in scientifically valid and methodologically sound evidence. In summary, this methodology section explicates the systematic procedures undertaken to gather and synthesize the relevant literature, underpinning the review’s objectives to chart AI’s transformative potential in psychiatry. The narrative review method, complemented by a stringent selection and synthesis process, ensures a comprehensive overview that highlights current innovations and delineates future research directions in the integration of AI technologies within psychiatric practices.

## 3. EEG and AI: New Horizons in Brain Wave Analysis

Electroencephalography (EEG) is a non-invasive method commonly used to measure brain activity and holds significant importance in neurological and psychiatric research due to its high temporal resolution [15]. EEG signals can map the detailed functions of the brain during various mental states, and based on these data, AI based models are being developed to detect disorders such as depression, schizophrenia, and bipolar disorder [16]. AI algorithms, when analyzing EEG signals, particularly utilize spectral features, band powers, and connectivity measures [17]. For example, findings related to low alpha and high beta powers derived from EEG signals are commonly used in the diagnosis of depression [18]. However, EEG data are susceptible to artifacts, necessitating controlled signal quality and the use of accurate data cleaning methods [19]. In particular, the removal of artifacts caused by eye movements and muscle activities enhances the accuracy of the analyses [20]. In recent years, deep learning techniques have played a significant role in disease detection and analyses to differentiate between brain activities using EEG data. For instance, in a study using deep neural networks, cases of treatment-resistant depression were classified with more than 90% accuracy compared to those responding to treatment [18]. Additionally, analyses of EEG signals from short-term and long-term recordings have been enhanced by functional connectivity analyses integrated with neuroimaging methods [21]. This section will thoroughly examine how EEG-based AI applications are utilized in areas such as emotional state detection, neurological disorder diagnosis, and treatment planning.

The studies summarized in this review reflect the significant advancements in EEG-based artificial intelligence applications for psychiatric and neurological research (see Table 1). These studies utilize a variety of ML and deep learning models, including CNNs, ensemble learning approaches, and advanced feature extraction methods such as wavelet scattering and functional connectivity analysis. This diversity in methodological approaches demonstrates the flexibility and adaptability of EEG signals for complex mental health evaluations.

One notable observation is the widespread use of CNN architectures in several studies. For instance, the GoogleNet CNN employed by Metin et al. achieved a classification accuracy of 90.05% for treatment-resistant depression (TRD) with a notable external validation accuracy of 73.33% [18]. Despite this success, the retrospective design and moderate sample size highlight the common limitation of data scarcity and generalizability concerns in psychiatric EEG research. Similarly, Xia et al.’s work utilized data augmentation techniques, such as discrete cosine transform (DCT), to enhance the performance of EEGNet models for sleep pattern analysis, achieving an accuracy of 92.85% [23]. This underscores the necessity of individualized data augmentation to improve model robustness. Moreover, studies focusing on ensemble methods, such as the research by Chen et al., illustrate the potential of combining multiple classifiers to increase diagnostic accuracy. Their ensemble model reached an impressive accuracy of 97.4% for ADHD detection based on EEG and behavioral measures, although the sample size was limited to 78 children, raising concerns about the representativeness of the findings [30]. In contrast, random forest classifiers, as seen in Earl et al.’s study on MDD, demonstrated promising accuracy across different emotional stimuli (e.g., happy and sad videos) [22]. However, the study’s reliance on a small cohort and the need for independent validation reveal common pitfalls in EEG studies, such as overfitting and the lack of demographic variability. Functional connectivity analysis has emerged as a pivotal feature in EEG-based studies of depression and anxiety disorders. The work by Lee et al. utilized phase-locking value (PLV) analysis in MDD patients and found no significant differences between self-harming and non-self-harming groups, suggesting the complexity of functional connectivity markers in behavioral phenotyping [40]. Similarly, Catal et al.’s analysis of intrinsic time scales linked EEG dynamics to behavioral modulation, although the limited assessment of individual variability constrained the study’s broader implications [43]. These findings highlight the potential of connectivity metrics but also underscore the need for more extensive multi-center datasets to improve model generalization. Interestingly, the integration of EEG with multimodal data, such as fMRI and behavioral assessments, was exemplified by Kung et al., who demonstrated the importance of neurovascular coupling through the spectral analysis of EEG-fMRI data [29]. Multi-omics approaches, as seen in Corrivetti et al.’s study, further indicate that combining EEG with biological samples can enhance personalized treatment predictions for MDD [26]. However, these methods introduce additional challenges related to data standardization and multi-site consistency. Another prominent challenge in EEG studies is the handling of artifacts and noise. Many studies, including those by Earl et al. and Cerdan-Martinez et al., employed bandpass filtering and independent component analysis (ICA) to address signal contamination [22,28]. Despite these preprocessing steps, the variability in artifact removal approaches underscores the need for standardized preprocessing protocols to ensure reproducibility across studies.

Finally, the review reveals the growing interest in brain–computer interface (BCI) applications. Jia et al.’s TTSNet model for EEG-based multi-class classification achieved a relatively modest accuracy of 45.88%, highlighting the complexity of designing robust BCI systems [48]. This result underscores the limitations of current neural network models in real-time cognitive state recognition and emphasizes the need for simpler, yet efficient, architectures to improve classification performance. In summary, this review underscores the significant strides made in EEG-based ML research across a range of psychiatric conditions, including MDD, ADHD, schizophrenia, and stress-related disorders. However, the field continues to face recurring challenges related to small sample sizes, model generalization, and data heterogeneity. Future research should prioritize the development of standardized preprocessing methods, robust multimodal datasets, and interpretable ML models to address these limitations. By overcoming these barriers, EEG-based AI applications can become more reliable and impactful in clinical practice.

## 4. ECG and AI: The Link Between Heart Rhythm and Mental Health

Electrocardiography (ECG) plays a crucial role in measuring the electrical activity of the heart and is widely utilized not only for the detection of cardiovascular disorders but also for assessing mental health conditions. ECG data, particularly heart rate variability (HRV) measurements, have broad applications in identifying stress, anxiety, and depression [49,50]. Given the impact of brain–heart connections in mental disorders, changes in heart rhythm are often considered biological representations of emotional states [49,51]. In recent years, significant advancements have been made in ECG signal analysis using deep learning and AI methods. For instance, one-dimensional convolutional neural networks (1D-CNNs) have demonstrated high accuracy in classifying stress and depression by processing raw ECG signals directly [50]. These end-to-end approaches simplify the data processing pipeline by reducing the need for traditional feature extraction steps [52]. Additionally, some studies have shown that short-duration ECG segments, recorded at different time intervals, can effectively detect mental health disorders [53,54]. Notably, the analysis of ECG data obtained from portable devices offers significant advantages for monitoring outside clinical settings [55].

AI-based ECG analyses have also been enhanced through multimodal approaches for stress detection. For example, combining ECG signals with respiration and skin conductance data enables more precise monitoring of stress and relaxation states [56,57]. In conclusion, the use of AI-driven ECG analysis has emerged as a reliable biomarker in mental health assessments. However, challenges such as inter-subject variability and signal noise necessitate the improvement and standardization of data processing methods [58,59]. This section will explore how AI-based ECG applications can be integrated into clinical and everyday mental health evaluations.

The studies presented in Table 2 highlight the growing role of electrocardiography (ECG) in mental health assessments, leveraging various ML and deep learning (DL) methodologies to detect psychiatric and emotional disorders. A key observation across these studies is the increasing focus on end-to-end learning approaches that eliminate the need for manual preprocessing. For instance, the pre-processing-free deep learning model proposed by Abedinzadeh et al. [59] achieved a remarkable 99.35% accuracy for mental state classification using raw ECG data, demonstrating the robustness of transfer learning techniques for noisy signal environments. However, limited validation for noise resistance in diverse conditions underscores the need for broader testing. In parallel, scalogram-based methods, such as those by Abbas et al. [52], showed the advantage of 2D representations of ECG and EEG data in depression detection, attaining high sensitivity (96%) and specificity (95%). These results highlight the importance of feature extraction through time-frequency transformations for multimodal signals. However, as noted in the study, the real-time implementation of Internet of Things (IoT) systems for remote monitoring may face stability issues in data transmission. Ternary pattern-based signal classification models, like the one introduced by Tasci et al. [51], further emphasize the significance of interpretable machine learning. By employing majority voting and feature selection, the model achieved an overall accuracy of 96.25% across multiple psychiatric conditions, including bipolar disorder and schizophrenia. Nevertheless, dataset-specific limitations and single-lead configurations may hinder the generalizability of this approach to multi-lead or more complex datasets. Multimodal fusion frameworks have gained traction in emotional health assessments, as seen in the CNN–LSTM hybrid model by Shermadurai et al. [60]. This approach, which integrated EEG, ECG, and accelerometer data, reported an impressive classification accuracy of 94.58% for stress detection. However, the increased dimensionality of multimodal data presents computational challenges that require optimization strategies to avoid overfitting and reduce resource demands. Models using wavelet scattering and cardiopulmonary coupling (CPC), such as Zhang et al.’s [61] ResAttNet framework, further demonstrate how signal processing techniques enhance the detection of mental workload changes. While these methods showed improvements over traditional HRV-based models, they often rely heavily on specific datasets like MAUS, highlighting the importance of testing across broader and more heterogeneous samples. Another notable contribution is the 1D-CNN-based framework for mental fatigue detection by Chen et al. [62], which achieved an impressive accuracy of 98.44%. However, the small sample size (22 participants) and the limited time windows for data collection may constrain the model’s applicability in larger-scale settings. Hybrid architectures combining deep learning and attention mechanisms, such as the framework proposed by Geethanjali et al. [63], are emerging as powerful tools for maternal health risk detection. Despite achieving 98.4% accuracy, the need for more diverse datasets remains a significant limitation to ensuring the robustness of these models across different demographics and clinical contexts. The inclusion of vision transformers in the CNN ensemble proposed by Waheed Awan et al. [64] for emotional health assessment illustrates the potential of state-of-the-art transformer models in physiological data classification. With an accuracy of 98.2%, this approach demonstrates the potential of combining different neural network architectures for improved generalization. However, the long training times and computational demands of transformers pose challenges for real-time and mobile health applications. Finally, fine-tuned feature extraction models such as Tuncer et al.’s [65] “Cardioish” framework provide an explainable artificial intelligence (XAI) solution for cardiac disorder classification with over 99% accuracy. This approach underscores the importance of interpretability in clinical diagnostics but also highlights the time-consuming nature of detailed feature extraction processes. The review of these ECG-based models reveals that while ML and deep learning approaches have made significant strides in mental and emotional health detection, there are still challenges related to data variability, dataset imbalances, and the generalizability of single-channel versus multi-channel data (Telangore et al. [66]). Additionally, models must be validated with diverse datasets and tested for real-world scenarios to overcome demographic and technical limitations. Future research should focus on developing lightweight, interpretable, and adaptive models that balance computational efficiency with diagnostic accuracy. Integrating multimodal data streams and exploring domain adaptation techniques may further enhance the reliability of ECG-based AI systems in mental health evaluations.

## 5. Speech Analysis and Artificial Intelligence: Detection of Emotional States

Speech analysis is a valuable tool for assessing individuals’ emotional and cognitive states as acoustic, prosodic, and linguistic features of speech often reflect underlying mental health conditions [72,73]. Acoustic features such as fundamental frequency, speech rate, energy intensity, and intonation, alongside prosodic features like pitch variation and sentence structure, provide critical insights into emotional states [74,75]. AI-based models, unlike traditional assessment methods, can process large speech datasets and deliver objective results while accounting for individual differences [76,77]. Recent studies have shown that deep learning frameworks significantly enhance the detection of emotional expressions through speech, achieving high accuracy rates [78]. This has made speech analysis an effective method for the early diagnosis of disorders such as depression, anxiety, and bipolar disorder [79,80]. Common indicators in speech patterns of individuals with depression include slower speech rates, monotonous tone, and prolonged pauses, which AI algorithms can accurately classify and support clinicians during the diagnostic process [81,82,83]. Moreover, remote assessments using voice logs and online conversations analyzed by AI models have demonstrated reliability in evaluating levels of depression, anxiety, and stress [84,85,86]. Speech-based emotional recognition systems have also proven effective in detecting conditions such as social phobia, schizophrenia, and post-traumatic stress disorder [87]. These systems can monitor shifts in emotional states during interactions between patients and healthcare providers, contributing to personalized treatment plans [88,89,90]. AI-driven speech analysis has also been integrated into mobile apps and online mental health support systems, enabling continuous monitoring of users’ emotional states and providing timely interventions [91,92]. The combination of AI with NLP has further enhanced the performance and accuracy of speech-based diagnostic tools [93,94]. However, challenges related to data privacy, ethical concerns, and transparency in AI decision-making processes must be addressed for widespread clinical adoption [95,96]. Additionally, the generalization of speech datasets across different cultural and linguistic contexts is essential for improving the robustness of these models [17,97].

In conclusion, AI-powered speech analysis holds significant promise for revolutionizing the assessment of emotional states and mental health conditions, and future advancements in data availability and model development are expected to further expand its clinical applications [98,99].

The research studies outlined in Table 3 highlight the growing integration of speech-based AI systems for detecting and classifying various mental health conditions. The use of both supervised and unsupervised learning models has demonstrated significant potential in identifying distinctive speech patterns related to psychiatric and neurological disorders. For example, Rezaii et al. [100] employed connected speech samples and a custom NLP classifier to classify variants of primary progressive aphasia (PPA) with an accuracy of 97.9%. However, their reliance on short speech samples raises concerns about capturing the full complexity of PPA-related speech disruptions. In schizophrenia research, large language models (LLMs) such as GPT and Llama have shown their capability in evaluating disorganized thought processes, achieving a notable 92% F1-score and demonstrating consistency comparable to expert ratings (Pugh et al. [101]). Despite this, a trade-off between the precision of the models and the interpretability of their outputs remains a challenge. Similarly, emotional speech recognition studies, such as the empirical analysis by Ahammed et al. [102], demonstrated exceptional classification accuracy across multiple datasets (e.g., 99.82% for TESS and 98.95% for SAVEE), leveraging Mel-frequency cepstral coefficients (MFCCs) and chroma features. Nevertheless, the lack of testing on larger real-world datasets limits the generalizability of these findings. Leite et al. [103] adopted an incremental learning approach for tracking bipolar disorder over a seven-month period, achieving an accuracy of 91.8% based on acoustic features such as pitch and energy. However, the overlapping classes in psychiatric speech data remain an obstacle, making it difficult to distinguish between different affective states. Additionally, Wang et al. [104] explored the use of semi-structured interviews and explainable AI (XAI) techniques for detecting loneliness in older adults, obtaining an accuracy of 88.9%. Despite achieving high recall scores, their study was constrained by a small gender-imbalanced sample, reflecting a broader issue in clinical AI research concerning demographic representation. In depressive disorder assessments, Park et al. [105] utilized speech samples from social media posts and classified depressive symptoms based on DSM-5 criteria. While this approach leveraged publicly available data, concerns about the reliability and authenticity of online speech content pose limitations for diagnostic accuracy. Furthermore, Ding et al. [106] used a multi-task deep learning model to analyze speech data from crisis hotline calls, achieving a 96% F1-score for suicide risk assessment. However, the small size of the dataset and the absence of multimodal inputs, such as video data, limit the robustness of their findings. Assistive technologies for visually impaired individuals, as explored by Rosi et al. [107], demonstrated the feasibility of combining speech and gesture recognition, achieving 96.3% accuracy using CNNs and OpenCV. However, limited real-world testing hinders the validation of these systems in dynamic real-life environments. Takeshige et al. [108] focused on Alzheimer’s disease detection through chatbot conversations, incorporating speech and facial feature extraction to distinguish Alzheimer’s patients from healthy participants with a 94% area-under-the-curve (AUC) score. Yet, the performance of such models remains highly dependent on the effectiveness of the chatbot interaction. Notably, the hybrid feature extraction approach by Taşcı et al. [109] demonstrated a 94.63% accuracy in detecting depression from speech audio signals by employing wavelet transforms and k-nearest neighbor (KNN) classification. While this model shows promise, further evaluation of larger and more diverse datasets is necessary to confirm its robustness across varied populations and settings.

Overall, these studies illustrate the versatility of speech-based AI models in the early detection and classification of various mental health conditions, including PPA, schizophrenia, depression, bipolar disorder, and Alzheimer’s disease. However, several challenges persist, such as the dependency on dataset quality, demographic biases, and the need for multimodal integration. Future research should focus on expanding dataset diversity, improving the interpretability of AI models, and enhancing robustness through longitudinal studies and multi-center trials. Additionally, ethical considerations regarding data privacy, transparency, and informed consent should be prioritized to ensure responsible implementation in real-world clinical settings.

## 6. Blood Tests and AI: New Approaches in Biomarker Analysis

The integration of AI in the analysis of blood-based biomarkers has revolutionized the understanding of the biological underpinnings of psychiatric disorders [110,111]. By processing complex and multidimensional data from blood samples, AI-based models can identify patterns linked to mental health conditions, such as depression, schizophrenia, and bipolar disorder, which are not discernible through traditional diagnostic methods [112,113]. These models utilize ML algorithms to analyze various biological markers, including inflammatory proteins, genetic expressions, and metabolic indicators, to classify and predict psychiatric disorders with enhanced precision [114,115]. One significant advancement is the use of multi-domain integration models that combine biomarkers from multiple sources, such as immune-inflammatory proteins and cognitive metrics, to improve the diagnostic differentiation between related psychiatric conditions [110]. Additionally, unsupervised clustering methods have enabled the discovery of subtypes within psychiatric disorders, offering more personalized treatment pathways [116,117]. The application of neural networks and advanced imaging of blood samples, such as scattergram images, has also emerged as a cost-effective method for detecting psychiatric conditions like schizophrenia with high classification accuracy [118,119]. Despite these advancements, challenges remain in the clinical validation and generalization of AI models across different populations and data sources [120,121]. The heterogeneity of psychiatric disorders requires large-scale studies and independent cohort validations to ensure robust and reproducible results [122,123]. Moreover, ethical considerations related to data privacy and the transparency of AI decision-making processes must be addressed to support the responsible implementation of these technologies in clinical settings [124,125].

In summary, the use of AI in the analysis of blood-based biomarkers represents a significant leap forward in precision psychiatry. By uncovering the molecular and genetic foundations of mental disorders, these approaches pave the way for more accurate diagnoses, early interventions, and personalized treatment plans [126].

## 7. Social Media and Psychiatry: A New Frontier in Mental Health Assessment

The increasing use of social media platforms has introduced novel avenues for understanding and addressing mental health challenges. Social media platforms such as Twitter, Facebook, and Reddit serve as rich sources of user-generated content, reflecting real-time emotional expressions, opinions, and health-related narratives [127,128]. These digital interactions provide valuable insights into public sentiment regarding psychiatric treatments and the lived experiences of individuals with mental disorders [129,130]. Studies leveraging ML and NLP have demonstrated that social media data can be used to predict and classify mental health outcomes, including depression, anxiety, and suicidality [131,132]. For example, ML models trained on social media conversations have achieved high levels of accuracy in detecting suicide risk factors and depressive narratives [133,134]. Additionally, social media analysis enables researchers to explore public perceptions of specific psychiatric interventions, such as antipsychotic medications, and identify misconceptions or concerns that may influence treatment adherence [135,136]. The COVID-19 pandemic has further highlighted the potential of social media as a tool for real-time mental health surveillance, with studies documenting shifts in anxiety levels and digital engagement patterns during lockdown periods [137,138]. However, challenges such as ethical considerations, privacy concerns, and the generalizability of findings across different cultural and linguistic groups must be addressed [139,140].

By combining AI-based tools with social media data, mental health professionals can gain a deeper understanding of the psychosocial impacts of global events, track public discourse on mental health, and develop targeted intervention strategies to support vulnerable populations [141,142]. Despite its promise, the integration of social media data into clinical practice requires robust validation and regulatory frameworks to ensure data integrity and user safety [132]. In this context, the following section will explore the potential of social media platforms as tools for early detection, intervention, and mental health promotion.

The utilization of large language models (LLMs) in the analysis of social media data for psychiatric research has emerged as a transformative approach, enabling more nuanced assessments of mental health conditions (see Table 4). Recent comparative studies, such as that by Gargari et al. [143], have demonstrated that LLMs like GPT-3.5 and GPT-4 outperform traditional NLP models in interpreting clinical cases based on DSM-5 criteria, although they face challenges with specific psychiatric disorders. This highlights the potential of LLMs for context-aware text interpretation while also emphasizing the importance of domain-specific fine-tuning. Similarly, Pugh et al. [101] explored thought disorder assessments in speech samples from schizophrenia patients using LLM-based models, revealing a trade-off between accuracy and consistency, indicating that while LLMs can capture complex linguistic patterns, their predictions can sometimes be inconsistent. A key strength of LLMs in psychiatry lies in their ability to process unstructured data from electronic health records (EHRs) and social media platforms. Turner et al. [144] utilized a semi-rule-based NLP pipeline for transdiagnostic psychiatry, achieving high classification accuracy (95–99%) across a large dataset of clinical notes (22,170 patients). However, the relatively low F1-scores (0.38–0.86) for certain labels suggest that nuanced clinical information may be lost in large-scale automated analyses. In contrast, studies like Botelle et al. [145], which employed fine-tuned BioBERT models for classifying text fragments related to interpersonal violence in mental health records, reported high precision and recall (89–98%). This underscores the value of pretrained transformer models in specialized tasks but also points to limitations in data diversity, which may hinder generalizability. In suicide risk prediction, LLM-based models have shown significant potential. Levis et al. [146] and Levis et al. [147] demonstrated that NLP-enhanced classification models applied to EHR notes from veterans identified high-risk individuals more accurately, with improvements in AUC scores (+19%). However, these models were constrained by their reliance on specific datasets, such as those from VA records, limiting their applicability to broader populations. Similarly, Tsui et al. [148] achieved an impressive AUC of 0.932 for predicting first-time suicide attempts using a combination of structured and unstructured data, but their results were influenced by historical data biases, indicating a need for more representative datasets. For speech-based mental health research, semantic analysis has proven effective in distinguishing psychosis-related speech markers. Studies like Çabuk et al. [149] and Arslan et al. [150] utilized POS tagging and SBERT-based embeddings to classify schizophrenia and schizophrenia spectrum disorders, achieving mean accuracies above 86%. These findings underscore the potential of LLMs in speech feature analysis for psychiatric disorders, although language dependency remains a challenge, as evidenced by their limited generalizability across non-English datasets. Additionally, Zaher et al. [151] demonstrated that narrative speech analysis could predict psychosis relapse within a two- to four-week timeframe, although large-scale validation remains necessary.

Overall, the integration of LLMs and NLP pipelines into social media and EHR analyses has advanced psychiatric research by improving the detection and prediction of mental health outcomes. However, as noted in studies by Kerz et al. [156] and Msosa et al. [167], there is a trade-off between model interpretability and predictive performance, particularly when explainable AI (XAI) methods are applied. Future research must address these limitations by incorporating diverse datasets, refining weak supervision techniques (e.g., Cusick et al. [171]), and developing robust frameworks for clinical validation. Such advancements will be essential to fully realize the potential of LLMs in enhancing mental healthcare delivery and suicide prevention efforts through social media analysis.

## 8. Open-Source Datasets for AI Applications in Psychiatry

The advancement of AI in the field of psychiatry is increasingly reliant on the availability and accessibility of diverse and high-quality datasets. These datasets are essential for training and validating AI models that are designed to understand, diagnose, and treat psychiatric disorders more effectively. As AI continues to permeate various aspects of psychiatric research, the need for open access to relevant data becomes paramount. This ensures that the development of AI tools remains transparent, reproducible, and ethical while also facilitating global collaboration among researchers. Table 5 provides a comprehensive overview of key open-source datasets that are pivotal for AI research in psychiatry. These datasets encompass a wide range of data types, including electroencephalogram (EEG), electrocardiogram (ECG), textual content, and multimodal information, reflecting the multifaceted nature of psychiatric conditions. The datasets listed have been curated for their utility in developing AI applications that can process complex biological, textual, and behavioral data in psychiatric settings. Each dataset is described with details about its features, data types, the total count of data points, and access links, providing researchers with vital resources to aid in their studies. By leveraging these open-source datasets, researchers can explore innovative approaches in psychiatric AI, such as developing predictive models for early diagnosis, enhancing patient monitoring through real-time data analysis, and personalizing treatment plans based on unique biomarkers. The ethical considerations, data quality, and standardization of these resources are also crucial for ensuring that AI applications in psychiatry are both scientifically valid and socially responsible.

## 9. Recent Advances and Future Trends in AI-Based Psychiatry

The field of AI in psychiatry is evolving rapidly, introducing innovative applications that enhance diagnostic accuracy, treatment personalization, and early intervention. In recent years, large language models (LLMs), NLP frameworks, and deep learning-based approaches have significantly improved the efficiency of clinical decision support systems. LLMs, with their capacity to process complex clinical narratives and diagnostic notes, have shown potential in identifying suitable candidates for advanced therapies, such as transcranial magnetic stimulation (TMS). Furthermore, multimodal approaches integrating data from electroencephalography (EEG) and functional magnetic resonance imaging (fMRI) have enabled more comprehensive analyses of neural activity patterns. These methods facilitate the identification of subtypes within psychiatric disorders, such as schizophrenia and bipolar disorder, by combining spatial and temporal neural information. Additionally, emotion recognition tools have advanced, supporting real-time mood tracking through mobile applications and wearable devices. By analyzing physiological data, such as heart rate variability and skin conductance, these AI-driven tools can provide real-time feedback on anxiety and stress levels, augmenting mental health monitoring.

A key trend for the future involves the enhancement of explainability in AI (XAI) models. Transparent and interpretable models are crucial for fostering trust among clinicians and patients, addressing ethical concerns, and improving usability in clinical settings. Moreover, the integration of diverse and multi-center datasets and international data-sharing networks is expected to enhance the generalizability of AI models across different cultural and demographic groups. Another promising avenue is the application of social media analysis in psychiatric research. By analyzing textual and audio data from social media platforms, researchers can identify early indicators of mental health crises, enabling the development of public health early-warning systems. However, this approach necessitates robust data privacy regulations and ethical frameworks to ensure responsible data usage. In summary, the use of AI in psychiatry holds great potential for improving early diagnosis, personalized treatment planning, and access to mental health services. Future advancements in data diversity, regulatory frameworks, and interdisciplinary collaborations will be essential for addressing existing challenges and unlocking the full potential of AI-driven innovations in mental healthcare.

## 10. Challenges and Limitations of AI in Psychiatry

The integration of AI into psychiatry presents several challenges and limitations that need to be addressed to fully harness its potential. One of the primary issues is data quality and availability. Psychiatric data are often heterogeneous, unstructured, and limited in size, which complicates the training of robust AI models. Comprehensive datasets that encompass diverse populations are essential to ensure reliability and accuracy across different clinical settings. Generalizability remains another significant concern as models trained on specific populations may underperform in different environments due to variations in language, culture, and demographics. Moreover, the interpretability of AI models remains a critical hurdle, particularly in the case of deep learning frameworks that function as opaque systems. The lack of transparency in such models can hinder their clinical adoption, making explainable AI (XAI) techniques indispensable for building trust among clinicians and patients.

Privacy, security, and ethical concerns surrounding sensitive psychiatric data also pose significant challenges. Ensuring compliance with data protection regulations and obtaining informed consent is vital to mitigate risks associated with AI deployment. Bias within AI models, inherited from training datasets, can perpetuate disparities in mental healthcare and reinforce existing inequalities. Effective measures to detect and mitigate bias are crucial to ensure fairness and equitable access to mental health services. Clinical integration poses another challenge as AI tools must fit seamlessly within healthcare workflows without creating additional burdens for clinicians. This requires user-friendly interfaces, comprehensive training programs, and supportive infrastructure. The validation and regulation of AI tools in psychiatry necessitate large-scale and multi-center studies to confirm their efficacy and ensure their generalizability. Regulatory frameworks must evolve to establish clear guidelines for the safe and ethical use of AI in psychiatric settings, ensuring that these technologies meet rigorous clinical standards.

## 11. Future Directions

Future research in the realm of AI and psychiatry should prioritize several key areas to fully harness the potential of AI technologies. First, there is a critical need to refine explainable AI (XAI) techniques. Enhancing the transparency of AI models will not only foster trust among clinicians and patients but will also facilitate regulatory approvals and integration into clinical practice. For instance, developing methods that can elucidate the decision-making processes of AI systems can help psychiatrists understand and validate the AI’s diagnostic and therapeutic recommendations. Additionally, the expansion of diverse datasets is crucial. Current AI models often suffer from biases that arise due to the homogeneity of the data on which they are trained. To address this, future efforts should focus on gathering and utilizing datasets that are representative of the global population, including varied demographics such as age, ethnicity, and socioeconomic status. This will help in developing AI systems that are effective and fair across diverse populations. Another vital area is the development of robust validated frameworks for clinical implementation. These frameworks should ensure that AI tools in psychiatry adhere to the highest standards of safety, reliability, and efficacy.

Collaboration between data scientists, clinicians, and regulatory bodies will be essential in creating guidelines and standards for the deployment of AI in clinical settings. Moreover, the integration of AI with emerging technologies such as genomics and neuroimaging could lead to groundbreaking advances in personalized psychiatry. For example, combining AI with genetic data could help predict individual responses to psychiatric medications, reducing the trial-and-error process in medication management. Ethical considerations must also be at the forefront of future research. As AI becomes more integrated into psychiatric care, researchers and practitioners must address issues related to privacy, consent, and the potential for AI to perpetuate or exacerbate inequalities in mental healthcare. Establishing ethical guidelines and conducting ongoing evaluations of AI applications will be essential to navigate these challenges. By addressing these challenges and limitations, AI has the potential to revolutionize psychiatric care, fostering more accurate diagnoses, enabling earlier interventions, and facilitating highly personalized treatment plans. The anticipated advancements could significantly enhance both the efficacy and efficiency of psychiatric services, ultimately leading to improved patient outcomes.

## 12. Conclusions

AI has emerged as a transformative tool in psychiatry, reshaping traditional diagnostic and treatment paradigms by analyzing complex biological, behavioral, and linguistic data. EEG-based AI applications have identified nuanced neural patterns linked to psychiatric disorders such as depression and schizophrenia, while ECG analyses leveraging heart rate variability have enhanced the detection of emotional and stress-related conditions. Similarly, speech analysis and NLP based techniques have enabled accurate assessments of cognitive and emotional states, advancing the detection and monitoring of thought disorders. The use of large language models (LLMs) has further expanded the potential of AI, supporting the interpretation of unstructured data for remote mental health monitoring. Emerging applications, such as AI-driven analyses of blood biomarkers, have opened new avenues for understanding the biological underpinnings of psychiatric disorders by integrating genetic, inflammatory, and metabolic data. Social media analysis has demonstrated potential for real-time mental health monitoring, offering valuable insights into population-level trends and early warnings for crises such as suicidality and anxiety surges.

Despite these advancements, significant challenges remain. The interpretability of deep learning models continues to hinder clinical adoption due to their opaque nature, while biases in training data pose risks of unequal outcomes. Additionally, the generalizability of AI models is often limited by the demographic composition of datasets, and privacy concerns surrounding sensitive psychiatric data present ethical and regulatory hurdles. To fully realize the transformative potential of AI in psychiatry, future research should prioritize expanding diverse and high-quality datasets, refining explainable AI (XAI) frameworks, and developing robust regulatory frameworks to ensure its safe and ethical deployment. Interdisciplinary collaboration between clinicians, data scientists, and policymakers is essential for addressing the current limitations and fostering innovation in the field. In conclusion, AI has the capacity to revolutionize psychiatric care by enhancing diagnostic accuracy, streamlining clinical workflows, and enabling personalized treatment strategies. By addressing existing limitations and maintaining ethical practices, AI-driven solutions can pave the way for a new era of precision psychiatry, ultimately improving mental health outcomes and accessibility for patients worldwide.

## Figures and Tables

**Table 1:** State-of-the-Art Studies on EEG-Based AI in Psychiatry.

Study	Methodology	Data Type	Investigated Disease/Condition	Sample Size	Preprocessing Type	Classifier	Limitations	Pros	Cons	Originality Point	Results
Metin et al. [18]	CNN (GoogleNet) and EEG	EEG signals	Treatment-resistant depression (TRD)	77 in the TRD group, 43 in the non-TRD group, and 40 in the control group	Z-score normalization	GoogleNet CNN	Moderate sample size and retrospective design	High classification accuracy for TRD detection	Moderate sample size and retrospective design	Use of GoogleNet CNN for TRD classification is innovative	TRD–non-TRD classification accuracy: 90.05%; external validation: 73.33%
Earl et al. [22]	Random Forest Classifier	EEG and functional connectivity measures	Major depressive disorder (MDD)	24 in the MDD group and 25 healthy controls	Bandpass filtering (0.5–45 Hz), artifact removal, and ICA	Random Forest	Small sample, need for independent validation, and age and gender differences	High accuracy across emotional states	Small sample size and limited generalizability	Multi-condition EEG analysis for MDD detection	Model 1: 92.3% accuracy (resting state), Model 2: 94.9% accuracy (happy video), Model 3: 89.7% accuracy (sad video); test accuracies: 60%, 80%, and 70%, respectively
Xia et al. [23]	Data Augmentation (DCT) and EEGNet	Sleep EEG signals	Sleep pattern analysis	-	Discrete cosine transform (DCT) and normalization	EEGNet (CNN)	Small sample size and need for individual data	Effective use of augmentation for performance improvement	Small sample size and limited generalizability	Individual-specific data augmentation for EEG signal analysis	Model accuracy reached 92.85%. Performance improved with individual-specific classification via data augmentation
Madhu et al. [24]	BiLSTM and Ensemble Learning	EEG signals	Psychological stress and meditation effects	69 students	Spectral analysis	BiLSTM-DT Ensemble	Limited data group and short observation post-meditation	Combines BiLSTM with ensemble methods for improved classification	Small sample size and limited observation duration	Innovative model for meditation-induced stress analysis	BiLSTM-DT model accuracy: 82%
Liu et al. [25]	Semi-supervised learning and CEEMDAN Fuzzy Entropy	Single-channel EEG signals	Fatigue detection (drivers)	-	CEEMDAN and bandpass filtering	Fuzzy Entropy + Self-Training	Single-channel EEG usage only	CEEMDAN method effectively improves classification accuracy	Single-channel EEG usage limits generalizability	Application of fuzzy entropy with self-training for EEG-based fatigue detection	RF classifier accuracy: 88.3%
Corrivetti et al. [26]	Multi-Omics Analysis and Opade AI Tools	EEG and biological samples	Major depressive disorder (MDD)	350 patients	Standardization	Random Forest + AI-Assisted Analysis	Multi-center data alignment challenges	Combines EEG and biological data for personalized treatment	Multi-center data alignment challenges	Application of multi-omics analysis for personalized psychiatry	Multi-omics data analysis and personalized treatment prediction
Cambay et al. [27]	QuadTPat and kNN	EEG signals	Stress detection	310 participants	CWNCA (Component analysis)	kNN	Complex model structure	High accuracy for stress detection models	Complex model structure	Innovative CWNCA-based analysis for stress classification	92.95% accuracy; LOSO cross-validation: 73.63%
Cerdan-Martinez et al. [28]	EEG Analysis and Hotelling’s T^2^	EEG signals	Watching violent films	30 students	Bandpass filtering	-	Small sample size	Detailed analysis of gender differences	Small sample size	Unique focus on EEG analysis during violent film exposure	Increased left insula activation in females
Kung et al. [29]	EEG-fMRI Spectral Analysis	EEG and fMRI data	Sleep inertia	-	Spectral filtering	-	Small sample size	Demonstrated neurovascular coupling in sleep inertia	Small sample size	Integrated EEG-fMRI spectral analysis	Neurovascular coupling state dependency was demonstrated
Chen et al. [30]	Ensemble Model and K-CPT-2	EEG and behavioral measures	Attention-deficit/hyperactivity disorder (ADHD)	78 children	Frequency band filtering	Ensemble Model	Small sample size	High accuracy for ADHD detection	Small sample size	Multimodal approach combining EEG and behavioral measures	Ensemble model accuracy: 97.4%
Maschke et al. [31]	EEG Dynamic Analysis and PCI	EEG signals	Loss of consciousness (effects of anesthesia)	-	-	-	Controlled experimental conditions	Effective EEG-based prediction of PCI values	Controlled experimental conditions	EEG dynamic properties applied to predict anesthesia outcomes	PCI values were predicted with EEG dynamic properties
Kim et al. [32]	Microstate Analysis	EEG signals	Internet gaming disorder and alcohol dependence	199 participants	Bandpass filtering	-	Single-session data collection	Identified distinct EEG microstate changes in AUD and IGD	Single-session data collection	Microstate analysis for co-occurring disorders (IGD and AUD)	Microstate C duration was different in AUD and IGD patients
Zhang et al. [33]	Wavelet Scattering and BiLSTM-MA	Multi-channel EEG	Major depressive disorder (MDD)	2 datasets	Wavelet transform	BiLSTM-MA	Dataset compatibility issues	Achieved high accuracy for MDD detection	Dataset compatibility issues	Novel use of wavelet scattering for EEG-based depression analysis	98.8% accuracy; F1-score: 99.81%.
Ouyang et al. [34]	EEG and PSG Analysis	EEG and sleep measurements	Brain aging and exercise	26 participants	Bandpass filtering	-	Small sample size	Observed cognitive improvements post-exercise	Small sample size	EEG and PSG combined to study aging and exercise effects	Cognitive performance improvement after exercise observed
Chen et al. [35]	EEG Functional Connectivity Analysis	EEG signals	Attention-deficit/hyperactivity disorder (ADHD)	78 children	Bandpass filtering	-	Limited age group	ADHD subgroups showed distinct connectivity patterns	Limited to a specific age group	Focused on subgroup analysis of ADHD using EEG functional connectivity	ADHD subgroups showed differences in connectivity models
Meinert et al. [36]	Co-Production Workshop	EEG and survey data	Epilepsy	-	-	-	Lack of participant diversity	Subcutaneous EEG found to be beneficial for epilepsy	Lack of participant diversity	Collaborative approach to assess SubQ EEG potential	SubQ EEG was found to be potentially beneficial
Hsu et al. [37]	Gamma Band Effective Connectivity	EEG signals	Mindfulness experiences	-	Gamma band filtering	SVM, Naive Bayes, and Decision Trees	Data scarcity	Decision tree model was highly accurate	Data scarcity	Focused on gamma band connectivity for mindfulness analysis	Decision tree accuracy: 91.7%
Gui et al. [38]	Neuroadaptive Bayesian Optimization	EEG signals	Social interaction	42 infants	Bandpass filtering	-	Limited age group	Observed differential responses to social cues	Limited to infants and small sample size	Neuroadaptive Bayesian optimization for EEG-based social cue analysis	Infants’ responses to social cues differed
Pandey et al. [39]	fNIRSNET Model and Multi-View CNN	fNIRS and EEG signals	Auditory event classification	9 participants	Standardization	CNN	Small sample size	Multimodal analysis combining fNIRS and EEG	Very small sample size	Novel CNN-based model combining fNIRS and EEG for auditory classification	87.15% accuracy achieved
Lee et al. [40]	FC Connectivity Analysis and PLV	EEG signals	Self-harm and depression	77 MDD patients	PLV analysis	-	Need for clinical validation	Explored differences in EEG functional connectivity	Need for clinical validation	First to study PLV differences in MDD with self-harm	No difference found between NSSI and non-NSSI groups
Zhang et al. [41]	Event-Related Potential (ERP) Analysis	EEG signals	Social anxiety disorder (SAD)	69 SAD patients	ERP component extraction	-	Need for control group	Observed ERP component changes after hypnotherapy	Lack of a control group	Focused on ERP-based analysis of hypnotherapy effects	N170 and LPP reductions observed after hypnotherapy
Ho et al. [42]	EEG Functional Connectivity and Graph Theory	EEG signals	Major depressive disorder (MDD)	54 MDD patients and 39 controls	Bandpass filtering	-	Variety of antidepressants used	Identified EEG functional connectivity differences	Variety of antidepressants used among participants	Graph theory applied to analyze EEG functional connectivity	Delta band EEG FC values found to differ
Catal et al. [43]	INT Change Analysis	EEG and calcium imaging	Brain time scales and behavior modulation	-	Bandpass filtering	-	Limited individual difference analysis	Correlated brain intrinsic time scales with behavior	Limited individual difference analysis	Used calcium imaging to investigate brain time scales	Brain intrinsic time scales correlated with behavior modulation
Moreau et al. [44]	EEG Hyperscanning	EEG signals	Autism spectrum disorder (ASD)	-	Spectral analysis	-	Small sample and limited interaction variety	Observed leadership behavior differences in ASD and TD	Small sample and limited interaction scenarios	Applied hyperscanning for leadership analysis in ASD	Different leadership behaviors observed in ASD and TD individuals
Dal Bo et al. [45]	ERP and Spectral Perturbation Analysis	EEG and ECG signals	Emotional cue effects	22 individuals (3 groups)	-	-	Small sample size	Analyzed olfactory sensory cue effects on EEG/ECG	Small sample size	Combined ERP and ECG analysis for emotional cues	Olfactory sensory cues showed significant ERSP analysis results
Yang et al. [46]	Efference Copy (EC) and CD Analysis	EEG signals	Schizophrenia and auditory hallucinations	-	Bandpass filtering	-	Small sample and schizophrenic patients only	Observed motor-sensory transformation differences	Small sample size and only schizophrenic patients	Efference copy analysis for auditory hallucinations in schizophrenia	Motor-sensory transformation differences observed in AVH and non-AVH groups
Thunstroem et al. [47]	Usability Testing	EEG signals	Mental health support tools	45 participants	-	-	Limited sample and healthy individuals only	Found chatbots more user-friendly than digital human interfaces	Limited sample and healthy individuals only	Usability study on EEG-guided mental health tools	Text-based chatbot was found to be more user-friendly than the digital human interface
Jia et al. [48]	TTSNet Model	EEG signals	Brain–computer interface (BC	-	Spectral analysis	CNN	Complex model	Applied multi-classification for BCI tasks	Complex model structure	Novel TTSNet for EEG-based multi-class BCI applications	TTSNet multi-classification accuracy: 45.88%

**Table 2:** State-of-the-Art ECG-Based Studies on Mental Health.

Study	Methodology	Data Type	Investigated Disease/Condition	Sample Size	Preprocessing Type	Classifier	Limitations	Pros	Cons	Originality Point	Results
Habib et al. [49]	MDDBranchNet and parallel-branch deep learning model	ECG	Major depressive disorder (MDD)	-	Signal segmentation	CNN and ANN	Limited to single-channel ECG for home use	Improved model accuracy for home-based applications	Limited to single-channel ECG for home use	Parallel-branch design tailored for MDD detection in low-resource environments	70% threshold showed consistent MDD detection; model accuracy increased by 7%
Abedinzadeh et al. [59]	Pre-processing-free deep learning with transfer learning	ECG	Mental state classification	-	No preprocessing	VGG16 and GoogLeNet	Limited noise resistance testing	Extremely high accuracy for noisy signals	Limited noise resistance testing	Avoids preprocessing while achieving state-of-the-art performance	99.35% accuracy for noisy ECG signals; VGG16 showed superior performance
Abbas et al. [52]	2D scalogram + MobileNetV2 + AdaBoost	EEG and ECG	Depression classification	2 datasets	Scalogram generation	MobileNetV2 and AdaBoost	IoT data transmission stability issues	Robust early detection and high sensitivity and specificity	IoT data transmission stability issues	Combination of scalogram and mobile-optimized deep learning for IoT systems	Sensitivity: 96%, Specificity: 95%, and MCC: 0.96; robust early depression detection
Tasci et al. [51]	Ternary pattern-based ANN with majority voting	ECG	Bipolar disorder, depression, and schizophrenia	3570 beats	Wavelet transform	ANN and Majority Voting	Dataset-specific model limitations	High overall accuracy for psychiatric disorders	Dataset-specific model limitations	Novel ternary pattern-based feature extraction	Overall accuracy: 96.25%; individual lead accuracy ranged from 73.67% to 89.19%
Shermadurai et al. [60]	CNN–LSTM hybrid model for multimodal analysis (EEG, ECG, and ACC)	EEG, ECG, and ACC	Stress classification	-	Kruskal–Wallis filtering	SVM, KNN, and RF	High-dimensional data increase computational costs	SVM achieved the best performance for multimodal stress analysis	High-dimensional data increase computational costs	Fusion of CNN and LSTM for multimodal stress classification	SVM achieved the highest performance with 94.58% accuracy
Zhang et al. [61]	Continuous wavelet transform (CWT) and cardiopulmonary coupling (CPC)	PPG	Mental workload classification	-	CPC mapping	ResAttNet	Limited to MAUS dataset	Improved over HRV-based methods	Limited to MAUS dataset	Use of CWT and CPC mapping for workload classification	80.5% accuracy; 6.2% improvement over HRV-based methods
Chen et al. [62]	1D-CNN-based mental fatigue detection	ECG	Mental fatigue	22 participants	Filtering	1D-CNN	Small sample size and limited to three time periods	High accuracy for mental fatigue detection	Small sample size and limited temporal data	Compact 1D-CNN design for mental fatigue analysis	Accuracy: 98.44%; F1-score: 98.44%
Geethanjali et al. [63]	Multimodal hybrid deep learning model for maternal health risk detection	Text and ECG	Maternal health risk analysis	-	Feature fusion	CNN, LSTM, and Attention	Requires more diverse datasets	High precision and recall for health risk detection	Requires more diverse datasets	Combined textual and ECG data for health risk prediction	Accuracy: 98.4%, precision: 97.6%, and recall: 95.6%
Waheed Awan et al. [64]	CNN–vision transformer ensemble with physiological signals (ECG, EEG, and GSR)	EEG, ECG, and GSR	Emotional health assessment	-	Signal segmentation	CNN, Vision Transformer, and SVM	Computationally intensive and long training times	High sensitivity and specificity for emotional assessment	Computationally intensive and long training times	Vision transformer-based ensemble for multimodal emotional health detection	Accuracy: 98.2%, sensitivity: 99.15%, and specificity: 99.53%
Sun et al. [67]	Feature fusion model with squeeze-excitation attention mechanism	ECG	Mental stress classification	-	PCA for dimensionality reduction	Voting Classifier	Model complexity increases computational demand	Improved performance over state-of-the-art models	Model complexity increases computational demand	Innovative attention mechanism to enhance feature fusion	Achieved improved accuracy compared to state-of-the-art methods
Mukherjee et al. [68]	Attention mechanism-based CNN–TLSTM for stress level detection	EEG and pulse rate	Stress level detection	-	Signal filtering	CNN and TLSTM	Limited validation with additional signals (ECG and GSR)	Robust classification across multiple stress categories	Limited validation with additional signals (ECG and GSR)	Attention-based TLSTM for multi-category stress detection	Average accuracy: 97.86%; robust classification into four stress categories
Sangeetha et al. [69]	Multimodal affective computing approach with neural network optimization	EEG, ECG, and EMG	Emotion recognition	-	Filtering and feature extraction	LSTM and CNN	Higher memory usage and training time	Reduced overfitting with neural network optimization	Higher memory usage and training time	Combined EEG, ECG, and EMG signals for emotion recognition	Highest accuracy: 87.83%; regularization reduced overfitting
Alzate et al. [70]	PPG and ECG feature extraction with ML framework	ECG and PPG	Depressive behavior detection	59 participants	Feature extraction	ML Models	Small sample size and requires further refinement	High accuracy for depressive state detection	Small sample size and requires further refinement	Integrated ECG and PPG signals for depression analysis	Accuracy: 92% for depressive state detection
Tuncer et al. [65]	Cardioish-based explainable feature extraction (XAI) model	ECG	Cardiac disorders	-	INCA and lead transformation	kNN	Lengthy feature extraction process	Over 99% accuracy across datasets	Lengthy feature extraction process	Explainable AI-based approach for cardiac disorder analysis	Over 99% accuracy for both datasets
Ao et al. [71]	Real-time cardiac abnormality detection using phase space–time delay	ECG	Cardiac abnormalities	-	Adaptive denoising	Automatic ML	Clinical validation is still needed	Accurate detection of APBs and P-wave peaks	Clinical validation is still needed	Phase space–time delay applied to real-time cardiac abnormality detection	APB detection accuracy: 100%; P-wave peak detection: 98.1%
Telangore et al. [66]	Wavelet scattering network (WSN) and machine learning	ECG	Bipolar disorder (BD), depression (DP), and schizophrenia (SZ)	3570 ECG beats	ECG signals segmented into 1-s epochs from 12-lead channels	Fine K-Nearest Neighbor (FKNN)	- Only male participants were included - The dataset is imbalanced (some classes have fewer samples) - Single-channel data may lead to loss of some signal features	Excellent accuracy across psychiatric disorders	Dataset imbalances and male participants only	Wavelet scattering for multi-class psychiatric disorder detection	Accuracy: 99.8%, precision: 99–100%, recall: 99–100%, and F1-score: 99–100% (tested with 10-fold cross-validation)

**Table 3:** State-of-the-Art Studies on Speech Analysis for Psychiatric Disorders.

Study	Methodology	Data Type	Investigated Disease/Condition	Sample Size	Preprocessing Type	Classifier	Limitations	Pros	Cons	Originality Point	Results
Rezaii et al. [100]	Cross-sectional study and supervised and unsupervised classification	Connected speech samples	Primary progressive aphasia (PPA)	78 patients	Feature extraction from speech	Custom NLP classifier	Limited to short speech samples	High classification accuracy for PPA variants	Limited to short speech samples	Combination of supervised and unsupervised techniques for PPA classification	97.9% accuracy in classifying PPA variants
Pugh et al. [101]	Model evaluation	Speech samples	Schizophrenia (thought disorder)	51 participants	Data parameter tuning	Large language models (GPT and Llama)	Trade-off between accuracy and consistency in outputs	High consistency comparable to expert ratings	Trade-off between accuracy and consistency	Application of LLMs for evaluating thought disorders	Consistency comparable to expert ratings; 92% F1-score
Ahammed et al. [102]	Empirical analysis	Emotional speech signals	Emotion recognition	RAVDESS, TESS, and SAVEE datasets	MFCC, Chroma, and Mel-spectrogram	SVM	Requires testing on larger datasets	Extremely high accuracy across multiple datasets	Requires testing on larger datasets	Use of diverse spectral features for emotion recognition	Accuracy: 99.82% (TESS) and 98.95% (SAVEE)
Leite et al. [103]	Incremental learning approach	Speech streams	Bipolar disorder	7 months of data	Feature ranking (Pearson–Spearman correlation)	eOGS	High class overlap in psychiatric data	Effective use of incremental learning over time	High class overlap in psychiatric data	Incremental learning for dynamic speech analysis in bipolar disorder	91.8% accuracy using 8 acoustic features
Wang et al. [104]	Proof-of-concept study	Semi-structured interviews	Loneliness in older adults	97 participants	LIWC feature extraction	Explainable AI (XAI)	Small sample size and gender imbalance	High recall and transparency in prediction models	Small sample size and gender imbalance	First study linking speech to loneliness using XAI	Accuracy: 88.9%, F1-score: 0.8, and recall: 1.0
Park et al. [105]	Multi-label classification	Social media speech	Depressive disorders (DSM-5)	Not specified	Label correlation analysis	Custom classifier	Reliability of online data questioned	Predictions aligned with DSM-5 criteria	Reliability of online data questioned	Multi-label classification for DSM-5-based depression assessment	Predictions based on DSM-5 criteria using speech features
Ding et al. [106]	Multi-task learning model	Crisis hotline speech data	Suicide risk assessment	Not specified	Gender-based feature extraction	Deep learning model	Small dataset and ignored multimodal data	High accuracy for identifying crisis speech	Small dataset and ignored multimodal data	Multi-task model for crisis speech analysis	F1-score of 96% in crisis recognition
Rosi et al. [107]	Experimental study	Speech and gesture recognition	Assistive tech for the visually impaired	Not specified	Image and audio feature extraction	CNN and OpenCV	Limited real-world testing	Accurate integration of speech and gesture for assistive tech	Limited real-world testing	Application of speech and gesture for assistive technologies	96.3% accuracy in hand and face recognition
Takeshige et al. [108]	ML model	Chatbot conversations	Alzheimer’s disease	192 participants	Facial and speech feature extraction	ML-based chatbot model	Dependency on chatbot performance	Effective use of chatbots for AD detection	Dependency on chatbot performance	Chatbot-based AD detection with facial and speech features	94% AUC in distinguishing AD from healthy controls
Taşcı et al. [109]	Hybrid feature extraction	Speech audio signals	Depression	MODMA dataset	Wavelet transforms and feature selection	KNN (k-nearest neighbor)	Needs evaluation on broader datasets	High accuracy for depression detection using MODMA dataset	Needs evaluation on broader datasets	Hybrid feature extraction for depression analysis in MODMA dataset	94.63% accuracy in depression detection

**Table 4:** State-of-the-Art Studies on Social Media for Mental Health Analysis.

Study	Methodology	Data Type	Investigated Disease/Condition	Sample Size	Preprocessing Type	Classifier	Limitations	Pros	Cons	Originality Point	Results
Gargari et al. [143]	Comparative study with LLMs	DSM-5 clinical cases	Psychiatric disorders	20 cases	RAG (retrieval augmented generation)	GPT-3.5, GPT-4, Aya, and Nemotron	Struggled with some specific disorders	GPT models outperformed other models	Struggled with some specific disorders	Comparison of multiple large language models for psychiatric diagnosis	GPT models outperformed others
Pugh et al. [101]	Thought disorder assessment	Speech samples from schizophrenia patients	Thought disorders	51 participants	Feature extraction	LLM-based models	Inconsistent predictions in some cases	High consistency with expert ratings and strong F1-score	Trade-off between accuracy and consistency in outputs	First application of LLMs for thought disorder assessment	Accuracy vs. consistency trade-off
Turner et al. [144]	NLP pipeline for psychiatry	EHR clinical notes	Transdiagnostic psychiatry	22,170 patients	Semi-rule-based pipeline	NLP-based classification	Limited by HDRS comparison	Comparable consistency with expert ratings	Accuracy vs. consistency trade-off	Use of LLMs for thought disorder assessment	Accuracy 95–99% (F1-score: 0.38–0.86)
Benger et al. [152]	Deep learning for MRI labeling	Brain MRI reports	Neurological abnormalities	5000 MRI reports	Binary and multi-class labeling	Report classifier	Reduced performance in non-expert labeling	High accuracy for binary labels	Reduced performance in non-expert labeling	Novel application of deep learning for MRI report labeling	High accuracy for binary labels
Wang et al. [153]	Sentiment-based NLP analysis	Hospital admission records	Delirium detection	3862 records	Sentiment feature extraction	ML-based model	Retrospective data limitation	Improved AUC with sentiment features	Retrospective data limitation	First integration of sentiment analysis for delirium detection	AUC: 0.930 (with NLP) vs. 0.869 (without NLP)
Levis et al. [146]	Suicide risk prediction	VA electronic medical records	Suicide prediction	5029 cases + controls	Unstructured text notes	ML classification	Lack of ensemble evaluation	Effective in identifying high-risk suicide groups	Lack of ensemble evaluation	Use of unstructured text for suicide risk classification	Top 10% risk group captured 29% of suicides
Botelle et al. [145]	Violence text classification	Mental health records	Interpersonal violence	3771 text fragments	BioBERT fine-tuned	Pretrained transformer	Limited data diversity	High precision and recall for violence classification	Limited data diversity	Application of BioBERT for interpersonal violence detection	Precision: 89–98% and recall: 89–97%
Ciampelli et al. [154]	ASR with semantic NLP	Speech transcriptions in schizophrenia	Schizophrenia	Not specified	Word error rate (WER) analysis	Random forest	ASR accuracy impact on NLP results	Accurate schizophrenia classification from speech	ASR accuracy impacts NLP results	Integration of ASR and semantic NLP for schizophrenia analysis	Accuracy: 76.7% (ASR) and 79.8% (manual)
Levis et al. [147]	NLP-enhanced risk prediction	Veterans EHR notes	Suicide risk prediction	4584 cases	Clinical note analysis	ML classification	Model limited to VA data	Improvement in AUC with NLP features	Limited to VA data	Combination of unstructured notes and structured data	AUC: 0.69 (+19% improvement)
Vaci et al. [155]	Clinical depression NLP extraction	UK-CRIS EHRs	Depression	Not specified	Active learning-based annotation	Statistical models	Lower performance for auxiliary variables	High accuracy for drug-related information extraction	Lower performance for auxiliary variables	First application of active learning for clinical depression NLP	High accuracy for drug-related information
Çabuk et al. [149]	Turkish speech feature analysis	Schizophrenia patient speech samples	Schizophrenia	76 participants	POS tagging and Word2Vec embeddings	K-means clustering	Language-dependent limitations	High classification accuracy for schizophrenia in Turkish speech	Language-dependent limitations	Application of POS tagging for schizophrenia analysis in Turkish	Accuracy: 86.84%
Zaher et al. [151]	Speech marker analysis	Psychosis-related speech data	Psychosis relapse prediction	Not specified	Narrative speech analysis	NLP-based approach	Lacks large-scale validation	Accurate prediction of relapse within 2–4 weeks	Lacks large-scale validation	Use of narrative speech markers for relapse prediction	Predicts relapse within 2–4 weeks
Arslan et al. [150]	Turkish SSD speech analysis	Turkish speech samples	Schizophrenia spectrum disorders	82 participants	SBERT-based features	Random forest	Limited generalizability across languages	Effective differentiation of SSD cases	Limited generalizability across languages	First use of SBERT-based features for SSD in Turkish speech	Mean accuracy: 86.8%
Kerz et al. [156]	Explainable AI (XAI) for mental health	Social media data	Mental health detection	Public datasets	Feature ablation and LIME explanations	BiLSTM and transformer	Trade-off between accuracy and interpretability	High interpretability of mental health prediction	Trade-off between accuracy and interpretability	Application of XAI for feature analysis in mental health models	Detailed feature interpretability
Sawalha et al. [157]	Sentiment analysis of PTSD data	AVEC-19 corpus speech data	PTSD detection	275 participants	Sentiment feature extraction	ML model	Small dataset	High accuracy for sentiment-based PTSD detection	Small dataset	Sentiment analysis for PTSD detection in AVEC-19 corpus	Balanced accuracy: 80.4%
Acosta et al. [158]	Sentiment analysis for mindfulness	CSQ-8 questionnaire texts	Mindfulness during COVID-19	154 responses	Transfer learning	Neural networks	Limited dataset size	High accuracy for mindfulness detection	Limited dataset size	First use of sentiment analysis for mindfulness assessment	Accuracy: 93.02% (first set) and 90.53% (second set)
Kizilay et al. [159]	Clinical high-risk for psychosis	CHR-P and HC speech samples	Clinical high-risk psychosis	107 participants	POS and semantic analysis	ML-based model	Small sample size	Effective differentiation between CHR-P and HC groups	Small sample size	Semantic and POS-based analysis for CHR-P detection	Accuracy: 79.6% and AUC: 0.86
Cox et al. [160]	Psychedelic therapy narrative analysis	Social media user narratives	Substance use reduction	1141 participants	Topic modeling	ML algorithms	Self-reported narrative bias	Identifies potential impact of therapy from narratives	Bias due to self-reported narratives	Use of topic modeling for analyzing psychedelic therapy narratives	Prediction accuracy: 65%
Irving et al. [161]	Psychosis risk prediction	South London and Maudsley EHRs	Psychosis risk	92,151 patients	LASSO-regularized Cox regression	ML risk calculator	Dataset limited to SLaM NHS Trust	High AUC for predicting psychosis risk	Dataset limited to SLaM NHS Trust	Large-scale application of LASSO-regularized regression	Harrell’s C: 0.85
Wu et al. [162]	Phenotype-mention identification	Free-text medical records	Phenotype embedding tasks	Not specified	Embedding-based adaptation	NLP-based pipeline	Model retraining required in new settings	High accuracy for phenotype extraction	Requires retraining for different datasets	Use of embedding-based methods for phenotype-mention identification	Accuracy: 93–97%
Horigome et al. [163]	Dementia detection through free conversation	Dementia patient conversations	Dementia diagnosis	432 datasets	Morphological analysis	ML classifier	Specific to outpatient clinic setting	Accurate dementia detection in outpatient settings	Specific to outpatient clinic settings	First application of conversational data for dementia detection	Accuracy: 90%, sensitivity: 88.1%, and specificity: 91.6%
Viani et al. [164]	Temporal extraction for psychosis	Mental health records	Duration of untreated psychosis	Not specified	Rule-based time expression	NLP pipeline	Complex annotation challenges	Accurate time normalization for psychosis diagnosis	Complex annotation challenges	Rule-based temporal extraction for psychosis diagnosis	Normalization accuracy: 71–86%
Tsui et al. [148]	Suicide attempt prediction	EHR structured + unstructured data	First-time suicide attempts	45,238 patients	NLP and ML feature extraction	Logistic regression and CNN	Historical data bias	High AUC for suicide prediction	Historical data bias	Combination of structured and unstructured data for suicide risk	AUC: 0.932
Oh et al. [165]	AD phenotype extraction	Alzheimer’s clinical notes	Alzheimer’s disease dementia	Not specified	Manual annotation for training	NLP pipeline	Limited to unstructured notes	High F1-scores for phenotype extraction	Limited to unstructured notes	Use of NLP pipelines for Alzheimer’s phenotype extraction	F1-score: 0.65–0.99
Arslan et al. [166]	Bipolar vs. psychosis speech analysis	Turkish speech samples	Bipolar disorder and psychosis	143 participants	Semantic similarity + POS analysis	ML classifier	Cross-sectional design	Effective differentiation of bipolar vs. psychosis groups	Cross-sectional design	Semantic and POS-based analysis for bipolar and psychosis detection	Accuracy: 82.45% (FEP vs. HC)
Msosa et al. [167]	Mental health crisis prediction	Mersey Care EHR	Depression-related crises	Large EHR dataset	MedCAT and BioYODIE annotations	LSTM and random forest	Psychiatrist feedback integration needed	High AUC for mental health crisis prediction	Psychiatrist feedback integration needed	Integration of MedCAT and ML for mental health crisis prediction	AUC: 0.901 (training) and 0.810 (test)
Furukawa et al. [168]	Cognitive restructuring in CBT	Japanese iCBT records	Depression	4369 records	T5-based NLP model	NLP-based prediction	Limited to Japanese records	High accuracy for restructuring prediction	Limited to Japanese records	Use of NLP for cognitive restructuring analysis in CBT	Accuracy: 73.5%
Kosowan et al. [169]	PTSD case identification	Pan-Canadian EMR	Post-traumatic stress disorder	12,104 patients	EMR free-text + diagnostic fields	NLP case definition	Diagnostic code variation by jurisdiction	High accuracy for PTSD identification	Diagnostic code variation by jurisdiction	First application of NLP case definition for PTSD in EMR data	Accuracy: 93.6%
Meerwijk et al. [170]	3ST suicide theory-guided NLP	Veteran progress notes	Veteran suicide risk	Not applicable	Domain-specific ontology	NLP-enhanced risk model	Ontology development challenges	Improved predictive accuracy	Ontology development challenges	Domain-specific ontology integration for suicide risk prediction	Improved predictive accuracy
Cusick et al. [171]	Weak supervision for suicidal ideation	Clinical notes	Suicidal ideation detection	17,978 notes	Rule-based weak supervision	CNN	Manual review for validation	High accuracy for suicidal ideation detection	Manual review for validation	Rule-based weak supervision for clinical suicide risk classification	Accuracy: 94% and F1-score: 0.82
Iqbal et al. [172]	ADE extraction pipeline	Psychiatric EHRs	Adverse drug events	264k patients	Semantic rule-based approach	ADE annotation pipeline	Contextual ambiguity challenges	High F1-score for ADE extraction	Contextual ambiguity challenges	Semantic rule-based pipeline for adverse drug event identification	F1-score: 0.83

**Table 5:** Key Open-Source Datasets for Psychiatry Research.

Dataset Name	Features	Data Type	Total Data Count	Access Link
MODMA (Multimodal Depression Analysis Dataset)	EEG (128-electrode and 3-electrode) and audio data	Multimodal (EEG and audio)	53 EEG (128-electrode), 55 EEG (3-electrode), and 52 audio Participants	http://modma.lzu.edu.cn/data/index/
DAIC-WOZ (Distress Analysis Interview Corpus)	Audio recordings, text, interview dialogues, and video	Audio, text, and video	621 interviews (face-to-face, teleconference, Wizard-of-Oz, and automated)	https://dcapswoz.ict.usc.edu/
IEMOCAP (Interactive Emotional Dyadic Motion Capture Database)	Audio, video, facial, and motion data	Multimodal (audio, video, and motion capture)	~12 h of 5 sessions, with 10 actors	https://sail.usc.edu/iemocap/
DEAP (Database for Emotion Analysis using Physiological Signals)	EEG, physiological signals, and facial expressions	EEG, physiological (GSR and EMG), and video	32 participants and 40 video/trials	https://www.eecs.qmul.ac.uk/mmv/datasets/deap/
SEWA (Automatic Sentiment Analysis in the Wild)	Video, audio, and text	Multimodal (video, audio, and text)	538 clips	http://sewaproject.eu/
Sentiment140	Tweet text	Text	1.6 million tweets	http://help.sentiment140.com/home
eRisk Dataset	Social media posts (text)	Text	800+ users and millions of posts	https://erisk.irlab.org/
AVEC 2019 (Audio/Visual Emotion Challenge)	Audio, video, and text (depression and emotion analysis)	Multimodal (audio, video, and text)	DAIC-WOZ, SEWA, and USoM corpora	http://avec2019.dbvis.de/
ABIDE I (Autism Brain Imaging Data Exchange)	Resting-state fMRI, structural MRI, and phenotypic data	Neuroimaging (fMRI and MRI)	1112 subjects (539 ASD and 573 control; ages 7–64)	http://fcon_1000.projects.nitrc.org/indi/abide/
COBRE (Center for Biomedical Research Excellence)	Resting fMRI, structural MRI, and phenotypic data	Neuroimaging (fMRI and MRI)	72 schizophrenia patients and 75 controls	https://fcon_1000.projects.nitrc.org/indi/retro/cobre.html
Psychiatry-ECG Dataset	ECG signals/beats for bipolar, depression, schizophrenia, and control groups	ECG signals	198 participants (62 bipolar, 17 depression, and 119 schizophrenia)	https://www.kaggle.com/datasets/buraktaci/Psychiatry-ECG

## Data Availability

Not applicable.
